# DOES SEXUAL ORIENTATION RELATE TO HEALTH AND WELL-BEING? A PROPENSITY-SCORE MATCHED ANALYSIS

**DOI:** 10.1093/geroni/igz038.1111

**Published:** 2019-11-08

**Authors:** Christi L Nelson, Ross Andel

**Affiliations:** University of South Florida, Tampa, Florida, United States

## Abstract

Around 2.7 million adults over the age of 50 self-identify as lesbian, gay, bisexual, and transgender (LGBT) in the United States. Past research suggests that additional stressors caused by being a socially stigmatized minority group can have a negative effect on health and well-being. The purpose of this study was to examine the relationships between sexual orientation and self-rated health, memory, and psychological well-being in a 1:3 propensity score-matched subsample from 2016 wave of Health and Retirement Study (HRS), a nationally representative study of older adults. Each lesbian/gay/bisexual (LGB) participant (n=140) was matched with three straight participants (n=420) on age, sex, and education. The average age was 53.8 years (SD=2.3 years), 54% were men, the average education was 14.3 years (SD=2.4 years). Logistic regression results indicated that LGB participants were almost twice as likely to report ever having depression (OR=1.85, 95% CI=1.23-2.80). Conversely, LGB participants were more likely to report having better health (OR=1.47, 95% CI= 1.04-2.07) than straight participants and the two groups did not differ significantly in memory (OR=1.16, 95% CI= 0.82-1.64) from their straight counterparts. In conclusion, it is possible that the stigma due to sexual orientation plays a role in psychological well-being but may also reflect in better physical health but not cognitive health. It is also possible that the better health in LGB participants reflects self-report bias.

